# Biomarkers of Oxidative Stress and Their Clinical Relevance in Type 2 Diabetes Mellitus Patients: A Systematic Review

**DOI:** 10.7759/cureus.66570

**Published:** 2024-08-10

**Authors:** Sabitha S, Shreelaxmi V Hegde, S Vinayak Agarwal, Delna NS, Ajita Pillai, Sheetal N Shah, Ramjeela S

**Affiliations:** 1 Department of Biochemistry, Srinivas Institute of Allied Health Sciences, Mangalore, IND; 2 Department of Biochemistry, Srinivas Institute of Medical Sciences and Research Center, Srinivas University, Mangalore, IND; 3 Department of Biochemistry, Rajiv Gandhi University of Health Science, Bengaluru, IND; 4 Pulmonary Medicine, Manipal Tata Medical College, Jamshedpur, IND; 5 Department of Paramedical Sciences, EMS Memorial Cooperative Hospital and Research Centre - College of Paramedical Sciences, Malappuram, IND; 6 Biological Sciences, DELBIODESK - Research and Innovations, Bhopal, IND; 7 Medicine, Baroda Medical College, Vadodara, IND; 8 Department of Pathology, Srinivas Institute of Medical Science and Research Center, Mangalore, IND

**Keywords:** diabetic education, types 2 diabetes, oxidative stress markers, systematic review, lipid peroxidation

## Abstract

Assessing oxidative stress is vital in managing type 2 diabetes mellitus (T2DM) and its complications. This systematic review aims to identify the most important oxidative stress markers in T2DM patients and predict associated complications. A literature search was conducted from 2013 to 2023, focusing on case-control, cohort, cross-sectional, and randomized control trials. The included studies had open access and scientific methodologies for assessing oxidative stress markers, while the excluded studies were not published in English or lacked primary objectives related to oxidative stress markers and T2DM or its complications. The quality of eligible studies was evaluated using the Newcastle-Ottawa Scale (NOS) for case-control, cohort, and cross-sectional studies and the Jadad Scale for RCTs. Eighteen studies were selected for the review and 25 potential markers like malondialdehyde (MDA), 11 thiobarbituric acid reactive substances (TBARS), 8-hydroxydeoxyguanosine (8-OHdG), glutathione (GSH), superoxide dismutase (SOD), and isoprostanes were found to be the most commonly used markers to assess oxidative stress in T2DM. These markers help to assess oxidative stress levels in T2DM individuals as well as correlate with diabetic complications. Therefore, assessment and understanding of the role of oxidative stress in T2DM pathophysiology are crucial for improving patient care and mitigating complications.

## Introduction and background

Oxidative stress is an imbalance between reactive oxygen species (ROS) and antioxidant defense mechanisms in the body. Chemically, ROS is a byproduct formed from hydrogen peroxide (H2O2), oxygen (O2), and water (H2O) [1,2]. In the human body, endogenous ROS are produced mainly from mitochondria as a byproduct of mitochondrial respiration [3].

Excessive ROS in the body exhausts cellular antioxidant defense mechanisms, leading to oxidative stress. This dysregulation of redox homeostasis has been linked to the pathogenesis of aging and various diseases, including diabetes [4,5]. Type 2 diabetes mellitus (T2DM) is a significant global health challenge, and its prevalence is increasing worldwide [6,7]. Studies have shown that oxidative stress is a key contributor to the pathogenesis and progression of T2DM and its associated complications [8].

These complications can be due to the damage caused by reactive oxygen species to mitochondrial DNA, proteins, and lipids [9]. In the body, ROS oxidize proteins, leading to altered protein structure and function, which alter their active sites and enzymatic activities, contributing to different disease conditions [10,11]. By directly attacking DNA, ROS can cause base modifications, strand breaks, and chromosomal rearrangements, leading to mutations and cell death and being an important cause of oncogenesis, which is, unfortunately, an under-recognized complication in long-standing diabetes [12-14]. Recent studies have increasingly recognized oxidative stress as a key contributor to the development and progression of diabetes and its complications. With the present evidence linking oxidative stress and T2DM pathology, there is a growing demand to identify potential biomarkers of oxidative stress for early detection, risk mitigation, and monitoring of patient responsiveness to medications.

The current systematic review aims to provide a qualitative summary of the diagnostic utility and predictive value of oxidative stress markers in the complications of T2DM. Given the abundance of literature on oxidative stress markers and diabetes, it is imperative to derive a qualitative analysis of the published literature to identify the most effective oxidative stress markers in T2DM. Therefore, this systematic review attempts to address the existing knowledge gaps and find the best oxidative stress markers in diabetic mellitus and its complications.

## Review

Materials and methods

**Figure 1:**
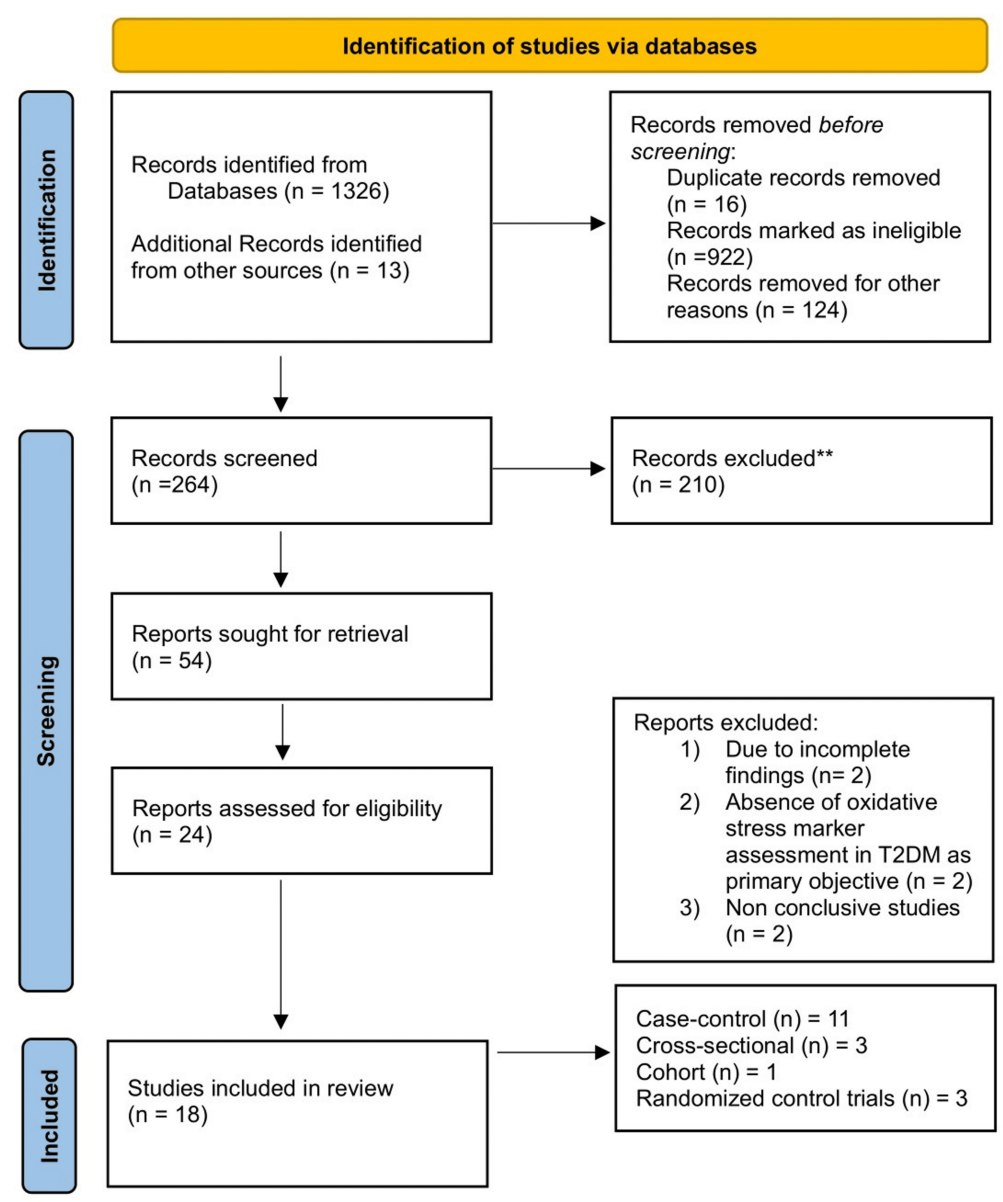
PRISMA flowchart explaining the selection of studies for systematic review. Reference [15].

For this systematic review, we adhered to the Preferred Reporting Items for Systematic Reviews (PRISMA) Updated Guidelines 2020 [15]. The article selection process is comprehensively illustrated in the PRISMA flowchart (Figure 1).

Search Strategy

A detailed literature search was conducted across scholarly databases such as PubMed, Google Scholar, and the Cochrane Library, with a focus on published studies between 2013 and 2023. Only case-control, cohort, cross-sectional, and randomized control studies employing systematic methodology for measuring oxidative stress were considered for review, and a detailed process of exclusion and inclusion was employed to filter the studies. Boolean operators such as ‘AND’ and ‘OR’ were utilized in the search, incorporating MeSH terms such as ('biomarker' OR 'biological marker' OR 'biomolecular marker' OR 'bioindicator*' OR 'biomolecular indicator') AND ('oxidative stress' OR 'oxidative damage' OR 'reactive oxygen species' OR 'ROS' OR 'oxidative biomarker' OR 'oxidative marker') AND ('type 2 diabetes mellitus' OR 'T2DM' OR 'diabetes mellitus, type 2' OR 'insulin dependent diabetes mellitus'). The search was limited to published, open-access studies from the last 10 years.

Eligibility Criteria

The eligibility criteria for this systematic review were precisely defined to ensure the inclusion of scientifically sound and relevant studies. The review considered only case-control, cohort, cross-sectional, and randomized control studies. Only fully open-access studies published between 2013 and 2023 were included. Studies that used detailed and scientific methodologies for oxidative stress marker assessment were included. Conversely, we excluded studies published in languages other than English and studies that did not assess oxidative stress markers or T2DM or its complications as a primary objective. Conference presentations were also excluded.

Data Management 

Data extraction and the preliminary reading of the study titles and abstracts were performed independently by the first, second, and third authors. The fourth and fifth authors then read the full text to check for eligibility. Any discrepancies that arose were resolved through discussion or consultation with the other authors. The data extracted from each eligible study were tabulated and included information such as the first author, year, study title, study design, sample size, oxidative stress markers analyzed, and conclusions of the study.

Quality Assessment

The quality of the selected studies was independently assessed by all authors using the Newcastle-Ottawa Scale (NOS) for case-control, cohort, and cross-sectional studies. The Jadad scale was used for randomized controlled trials (RCTs). The NOS for case-control and cohort studies evaluated criteria such as the adequacy of the case definition, representativeness of cases, selection and definition of controls, comparability of cases and controls, ascertainment of exposure, and non-response rate.

For cross-sectional studies, the NOS quality evaluation assessed the representativeness of the sample, sample size, selection of study subjects, comparability between groups, ascertainment of exposure, assessment of outcome, and description of statistical tests. Quality evaluation with the Jadad scale for RCTs assessed randomization, blinding, and handling of withdrawals and dropouts.

Results

Search Results

The PRISMA flow diagram (Figure 1) depicts the process for selecting appropriate studies for review. We conducted a systematic search utilizing MeSH terms, Boolean operators, and logical operators, which yielded 1339 records. After applying suitable filters and limiting the selection to studies published between 2013 and 2023, a team of four authors, with additional assistance, screened and sorted the list to select 54 studies for further evaluation. Out of the 54 studies, 24 met the criteria, including case-control, cohort, cross-sectional, and randomized control studies. After reading the full texts, six studies were excluded, leaving a total of 18 studies that fulfilled the inclusion criteria (Table 1).

**Table 1:** Describes the characteristics of each study, including study design, sample size, oxidative stress markers assessed, and key findings. References [16-34]. LPO: lipid hydroperoxide, NO: nitric oxide, TAC: total antioxidant capacity, GPx: glutathione peroxidase, NPDR: non-proliferative diabetic nephropathy, AGEs: advanced glycation end products, TBARS: thiobarbituric acid reactive species, HO: haem oxygenase, 8-OHdG: 8-hydroxydeoxyguanosine, MDA: malondialdehyde, POVPC: 1-palmitoyl-2-[5-oxovaleroyl]-sn-glycero-3-phosphorylcholine, PGPC: 1-palmitoyl-2-glutaroyl-sn-glycero-3-phosphorylcholine, SOD: superoxide dismutase, GSH: reduced glutathione, CAT: catalase, AOPP: advanced oxidized protein product, TBARS: thiobarbituric acid reactive substances, MetS: metabolic syndrome, AMI: acute myocardial infarction, TOS: total oxidant status, TAS: total antioxidant status, IMA: ischemia-modified albumin, OSI: oxidative stress index, NAFLD: nonalcoholic fatty liver disease, NASH: nonalcoholic steatohepatitis, PTGS2: prostaglandin-endoperoxide synthase 2, GST: glutathione S-transferase activity, HEL: Nε (hexanoyl)lysine, 15-F2t-IsoP: 8-iso-prostaglandin F2α, or 8-epi-prostaglandin F2α.

First author, year	Study title	Study design	Sample size	Oxidative markers assessed	Findings
Rodríguez-Carrizalez et al. [16]	Oxidants, antioxidants, and mitochondrial function in non-proliferative diabetic retinopathy [NPDR].	Transverse analytical study	270 T2DM patients + healthy, sex, and age-matched controls.	MDA	MDA is high in T2DM+NPDR patients.
				NO	NO levels increase along with the severity of retinopathy.
				TAC	TAC increases in T2DM with NPDR.
				CAT	Erythrocyte catalase activity increases in T2DM+NPDR patients.
				GPx-activity of erythrocytes	GPx activity increases in T2DM+NPDR patients.
Andrews Guzmán et al. [17]	Glycemic control and oxidative stress markers and their relationship with the thioredoxin interacting protein (TXNIP) gene in type 2 diabetic patients	Case-control study	20 normal weight T2DM + 20 T2DM Obese + 20 controls	TBARS	TBARS increased in T2DM+obese and normal-weight subjects.
				8-Isoprostanes	8-Isoprostanes increased in obese T2DM patients.
				HO	HO increased only in normal-weight T2DM subjects.
				AGES	AGES increased in T2DM+obese and normal-weight subjects.
Ono et al. [18]	Association of coronary artery calcification with MDA-LDL-C/LDL-C and urinary 8-isoprostane in Japanese patients with type 2 diabetes.	Observational cross-sectional study	75 T2DM Men and 88 T2DM Females	Urinary 8-isoprostane	Urinary 8-isoprostane and serum MDA are high in the coronary artery calcification group
				8-OHdG	No significant difference in urinary 8-OHdG of the coronary artery calcification groups 1 and 2.
				MDA	MDA is high in the coronary artery calcification group.
Eftekhari and Akbarzadeh [19]	The effect of calcitriol on lipid profile and oxidative stress in hyperlipidemic patients with T2DM	Double-blind randomized placebo-controlled trial	35 T2DM male+ 35 T2DM females	MDA	MDA levels decreased in both treatment and placebo groups.
Vinetti et al. [20]	Supervised exercise training reduces oxidative stress and cardiometabolic risk in adults with T2DM	Randomized controlled trial	20 T2DM male subjects aged 40-70	POVPC	Subjects with T2DM present a more oxidizing environment than healthy subjects.
				PGPC	Subjects with T2DM present a more oxidizing environment than healthy subjects.
Okoduwa et al. [21]	Age-dependent alteration of the antioxidant defense system in hypertensive and T2DM patients	Case-control study	Total 200 T2DM subjects [50 T2DM+ 50 hypertensives+ 50 T2DM hypertensive+ 50 nondiabetic normotensives]	MDA	Lipid peroxidation is high in the diabetic group.
				GSH	-
				SOD	SOD decreased in all groups.
				CAT	CAT decreased in all groups.
Brzović-Šarić et al. [22]	Levels of selected oxidative stress markers in the vitreous and serum of diabetic retinopathy patients	Case-control study	40 T2DM subjects [10 PDR+10 NPDR+ 10 T2DM+ 10 control]	LPO	LPO activity is significantly higher in patients with PDR.
				SOD	SOD activity is significantly higher in patients with PDR.
				MDA	MDA activity is significantly higher in patients with PDR.
				GSH	No difference in GSH between diabetic retinopathy patients and controls.
				AOPP	No significant difference in AOPP between patients and controls.
Hasan and Mohieldein [23]	Effects of tocotrienol-rich fraction supplementation on lipid profile and oxidative status in T2DM: a crossover-controlled trial	A randomized double-blinded placebo-controlled crossover trial	32 T2DM patients	MDA	Supplementation with TRF significantly reduced MDA levels
				8-OHdG	TRF supplementation significantly reduced 8-OHdG levels.
				SOD	SOD activity was not significantly different between TRF and placebo.
Ali et al. [24]	Correlation of red cell distribution width with oxidative stress in T2DM patients	Case-control study	100 T2DM patients	CAT	No significant difference in CAT between patients and controls.
				GSH	Serum GSH significantly decreased in T2DM patients compared to controls.
Abd El-Kader et al. [25]	Impact of weight loss on oxidative stress markers and TNF-α in obese type 2 diabetic patients	Clinical trial	25 T2DM males+ 25 T2DM females	MDA	Serum MDA decreased significantly after weight loss.
				CAT	Serum CAT activity increased significantly after weight loss
				SOD	Serum SOD activity increased significantly after weight loss.
				GPx	Serum GPx activity increased significantly after weight loss.
Casoinic et al. [26]	Involvement of oxidative stress in patients with T2DM and cardiovascular disease	Case-control study	47 T2DM patients with CVD	Urinary 8-isoprostane	Urinary 8-isoprostane levels were significantly higher in patients with T2DM+CVD than in healthy controls.
				MDA	MDA levels were significantly higher in patients with T2DM+CVD than in healthy controls.
Spanidis et al. [27]	The association of redox status with the severity of diabetic retinopathy	Cross-sectional study	172 T2DM patients [89 NPDR + 54 PDR + 29 non-DR]	GSH	GSH is significantly reduced in NPDR and PDR patients.
				Plasma protein carbonyls	Plasma protein carbonyls significantly increased in NPDR and PDR patients.
				TAC	TAC significantly decreased in NPDR and PDR patients.
				TBARS	TBARS levels significantly increased in NPDR and PDR patients.
				sORP	sORP significantly increased in NPDR and PDR patients.
				CAT	CAT levels decreased in NPDR and PDR patients.
				NO	NO levels increased in NPDR and PDR patients.
Inci et al. [28]	Relationship between total antioxidant status, total oxidant status, and oxidative stress index in patients with type 2 diabetes	Case-control study	90 T2DM patients + 30 control subjects	TAS	TAS significantly decreased in T2DM patients.
				TOS	TOS significantly increased in T2DM patients.
				OSI	OSI significantly increased in T2DM patients.
				IMA	IMA significantly increased in T2DM patients.
Alrefai et al. [29]	Circulating oxidized LDL is a useful marker for identifying silent myocardial ischemia in asymptomatic type 2 diabetic patients	Case-control study	98 T2DM patients	MDA	MDA and ox-LDL levels were significantly higher in patients with silent myocardial ischemia.
				Ox-LDL	MDA and ox-LDL levels were significantly higher in patients with silent myocardial ischemia.
Arab and Steghens [30]	Effects of combination therapy with lipoic acid and genistein on oxidative stress and lipid profile in type 2 diabetes patients: a randomized, double-blind placebo-controlled clinical trial	Double-blind placebo-controlled clinical trial	44 T2DM patients	MDA	MDA and ox-LDL levels were significantly higher in patients with silent myocardial ischemia.
				TAC	TAC significantly increased in the lipoic acid and genistein combination therapy group.
				GPx	GPx activity significantly increased in the lipoic acid and genistein combination therapy group.
				TOS	TOS significantly decreased in the lipoic acid and genistein combination therapy group
Mallard et al. [31]	Impact of intermittent fasting on oxidative stress markers and glycemic control in patients with type 2 diabetes	Clinical trial	50 T2DM males + 50 T2DM females	MDA	MDA levels significantly decreased in the intermittent fasting group.
				GPx	GPx activity significantly increased in the intermittent fasting group.
				TAC	TAC significantly increased in the intermittent fasting group.
				Urinary isoprostane-8-iso PGF2α	Urinary isoprostane-8-iso PGF2α levels significantly decreased in the intermittent fasting group.
Pouvreau et al. [32]	Mitochondrial DNA damage in early-onset type 2 diabetes is not associated with altered mitochondrial function or oxidative phosphorylation	Cross-sectional study	45 early-onset T2DM patients	8-OHdG	Mitochondrial 8-OHdG levels were significantly higher in early-onset T2DM patients.
				Urinary isoprostane-8-iso PGF2α	Urinary isoprostane-8-iso PGF2α levels were significantly higher in early-onset T2DM patients
Kahal et al. [33]	Effect of induced hypoglycemia on inflammation and oxidative stress in type 2 diabetes and control subjects	Case-control study	10 T2DM patients + 8 control subjects	FGF8	FGF8 was elevated and differentiated T2DM from controls. The elevation in FGF8 may suggest activation of protective mechanisms, as FGF8 has been shown to inhibit oxidative stress.
Rani and Mythili [34]	Lipid peroxidation and its correlation with antioxidants and lipoproteins in type 2 diabetes mellitus	Case-control study	200 T2DM subjects	MDA	MDA levels were significantly higher in T2DM subjects compared to controls.
				TAS	TAS levels were significantly lower in T2DM subjects compared to controls.

Study Characteristics

A total of 18 studies were selected for final review. The selected studies consisted of 11 case-control studies, 1 cohort study, 3 cross-sectional studies, and 3 randomized controlled trials. The characteristics of each study, including study design, sample size, oxidative stress markers assessed, and key findings, are summarized in Table 1.

Different markers have been used in studies to measure oxidative stress in T2DM patients. The most common markers used in the majority of the studies were malondialdehyde (MDA) and superoxide dismutase (SOD), followed by isoprostanes, reduced glutathione (GSH), catalase (CAT), total antioxidant capacity (TAC), glutathione peroxidase (GPx), 8-hydroxydeoxyguanosine (8-OHdg), lipid peroxidation (LPO), thiobarbituric acid reactive substances (TBARS), total oxidant status (TOS), and total antioxidant status (TAS).

**Figure 2:**
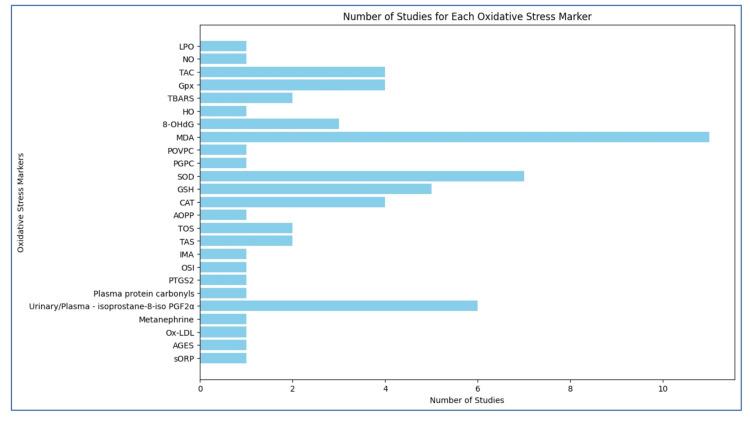
Bar chart depicting the frequency of different oxidative stress markers analyzed in studies reviewed, highlighting the number of studies conducted for each marker. MDA was the most common marker used by majority of the studies.

Markers such as nitric oxide (NO), heme oxygenase (HO), and 1-palmitoyl-2-[5-oxovaleroyl]-sn-glycero-3-phosphorylcholine (POVPC), 1-palmitoyl-2-glutaroyl-sn-glycero-3-phosphorylcholine (PGPC), advanced oxidized protein product (AOPP), ischemia-modified albumin (IMA), oxidative stress index (OSI), prostaglandin-endoperoxide synthase 2 (PTGS2), plasma protein carbonyls, metanephrine, ox-LDL, advanced glycation end products (AGEs), and sORP were not measured in more than one study (Figure 2).

Discussion 

There are diverse methodologies employed by researchers for assessing oxidative stress markers. This oxidative stress assessment is highly important for the treatment of various diseases. They can act as crucial indicators of cellular damage and provide information about the pathophysiological mechanisms of diseases such as diabetes, cancer, and metabolic syndromes. Several mechanistic and epidemiological studies point to the connection between diabetes and cancer through ROS [35].

Type 2 diabetes is a chronic metabolic disorder characterized by insulin resistance and relative insulin deficiency [34]. Oxidative stress plays a significant role in the pathophysiology of T2DM, contributing to its development and progression, as well as the associated complications [36]. Several oxidative stress markers have been associated with T2DM. The important marker discussed in the majority of the studies is MDA, which is a byproduct of lipid peroxidation that is frequently elevated in T2DM patients and indicates increased oxidative damage to lipids, which contributes to the development of complications. Furthermore, alterations in antioxidant defense mechanisms, such as decreased activity of SOD, catalase (CAT), and glutathione peroxidase (GPx), are frequently reported in T2DM patients, further exacerbating oxidative stress.

The most important markers of oxidative stress identified through the review are discussed in brief below.

MDA

Malondialdehyde is a product of polyunsaturated fatty acid peroxidation. Lipid peroxidation occurs when free radicals attack the carbon-carbon double bond of lipids. An increase in free radicals in the body is reflected by an increase in MDA levels [37]. Although there are variations in the levels of MDA in different samples, MDA is considered a reliable marker of oxidative stress under various disease conditions [38]. The thiobarbituric acid (TBA) assay is commonly employed to measure MDA in biological samples. Due to the instability of MDA in biological samples and the lack of specificity of the TBA reaction with MDA, total TBARS are measured as an oxidative stress biomarker in many studies [38].

SOD

Superoxide dismutase is an essential metalloenzyme found in living organisms. It serves as the first line of defense against reactive oxygen species. There are various isoforms of SOD based on their metal cofactors, as described in previous studies [39]. SOD can spontaneously dismutate superoxide radicals and cleave hydrogen peroxides and hydroperoxides to create stable molecules by undergoing the oxidation-reduction of metal ions [40].

CAT

Catalase is another key enzyme in the first line of defense against reactive oxygen species and is an important NADPH-binding protein [41]. It protects the body from oxidative damage by converting hydrogen peroxide into water and oxygen. CAT and SOD are enzymes involved in the same pathway of free radical mitigation [40-43].

GSH

The ratio of reduced GSH to GSSG is an indicator of cellular oxidative stress levels [44]. Glutathione disulfide is an important redox couple in cells, and its deficiency causes oxidative stress in the body. Hence, GSH is involved in the pathogenesis of various diseases [45].

Isoprostanes

These are a type of prostaglandin-like compound formed independently of the cyclooxygenase pathway and generated by the nonenzymatic peroxidation of PUFAs, especially arachidonic acid [45]. Arachidonic acid peroxidation produces three arachidonoyl radicals that undergo endocyclization to form four PGH2-like bicyclic endoperoxides [46]. Under oxidative stress, a fraction of lipid hydroperoxides undergo rearrangement and cyclization reactions to form isoprostanes [45,47]. Isoprostanes are stable compounds that can be detected in various biological samples, such as urine, plasma, serum, and tissues [45]. Isoprostanes can be categorized into several types based on their structural features and the position of oxygenation on the arachidonic acid molecule. Among them, F2-isoprostanes (F2-IsoPs) are the most abundant and well-studied class of isoprostanes, followed by F4-neuroprostanes, E2-isoprostanes, D2-isoprostanes, and isoflavanoids [46,48,49].

GPx

Glutathione peroxidase utilizes GSH as a cofactor in its catalytic cycle. In the presence of reduced GSH, GPx catalyzes the reduction of peroxides to their corresponding alcohols, forming oxidized glutathione (GSSG) in the process. The reaction mechanism typically involves the transfer of electrons from GSH to the peroxide substrate, resulting in the formation of water or alcohol and the oxidation of GSH to GSSG [50,51].

8-OHdg

8-Hydroxydeoxyguanosine is a biomarker of oxidative DNA damage [52]. ROS can directly attack DNA, and guanine nucleotides are easily oxidized to 8-oxo-7,8-dihyroguanine due to their low redox potential [53,54]. The formation of 8-OHdG leads to mutations in the DNA sequence if not repaired spontaneously. The accumulation of 8-OHdG in the genome is associated with various pathological conditions [55]. Apart from other oxidized guanine molecules, 8-OHdg can cross the cell membrane. Hence, the presence of 8-OHdg can be detected in bodily fluids such as urine and serum, and 8-OHdg can be used as a sensitive marker for oxidative stress assessment [56]. Measurement of 8-OHdG in urine and serum samples can indicate the progression and development of complications in diseases such as diabetes, cancer, and some bacterial infections [22,32,57,58].

Although we followed a strict systematic methodology to filter out the best oxidative stress markers, there are some limitations in the current review that need to be considered. First, the heterogeneity among studies in terms of sample size, study design, and methodology may introduce variability in the findings, which limits the generalizability of the results. Additionally, many of the reviewed studies relied on cross-sectional and case-control study designs, which prevent the establishment of causal or temporal relationships between oxidative stress markers and diabetic complications. Therefore, longitudinal studies with larger cohorts are necessary to validate the predictive utility of these markers and better understand their role in disease progression.

## Conclusions

This systematic review provides a qualitative summary of oxidative stress markers to predict complications in type 2 diabetes patients. Lipid peroxidation is the most common indicator of oxidative damage and is measured by assays such as TBARS and MDA levels. The levels of specific oxidative stress biomarkers, such as 8-OHdG, can be measured in both urine and serum. These markers provide insights into pathophysiological mechanisms and serve as potential prognostic indicators for disease progression and therapeutic response. Measuring oxidative stress markers can help mitigate complications in T2DM. The majority of the studies measured MDA as an oxidative stress marker, followed by SOD> Isoprostanes>GSH>TAC>Gpx>CAT>TOS>TAS>TBARS. Other important markers of oxidative stress measurement were LPO, NO, HO, POVPC, PGPC, AOPP, IMA, OSI, PTGS2, protein carbonyls, Ox-LDL, AGES, and sORP.
